# Epigenetic inactivation of *SLIT3* and *SLIT1* genes in human cancers

**DOI:** 10.1038/sj.bjc.6602222

**Published:** 2004-11-09

**Authors:** R E Dickinson, A Dallol, I Bieche, D Krex, D Morton, E R Maher, F Latif

**Affiliations:** 1 Section of Medical and Molecular Genetics, Division of Reproductive and Child Health, Institute of Biomedical Research, University of Birmingham, Birmingham B15 2TT, UK; 2 Laboratoire d’Oncogénétique – INSERM E0017, Centre René Huguenin, 35, rue Dailly, F-92210 St-Cloud, France; 3 Department of Neurosurgery, Universitätsklinikum Carl Gustav Carus, Technische Universität Dresden, Fetscherstraße 74, 01307 Dresden, Germany; 4 Department of Surgery, University of Birmingham, Birmingham B15 2TT, UK; 5 Cancer Research UK Renal Molecular Oncology Research Group, University of Birmingham, Birmingham B15 2TG, UK

**Keywords:** *SLIT1*, *SLIT3*, methylation, tumours

## Abstract

In Drosophila, the Slit gene product, a secreted glycoprotein, acts as a midline repellent to guide axonal development during embryogenesis. Three human Slit gene orthologues have been characterised and recently we reported frequent promoter region hypermethylation and transcriptional silencing of *SLIT2* in lung, breast, colorectal and glioma cell lines and primary tumours. Furthermore, re-expression of *SLIT2* inhibited the growth of cancer cell lines so that *SLIT2* appears to function as a novel tumour suppressor gene (TSG). We analysed the expression of *SLIT3* (5q35–34) and *SLIT1* (1q23.3–q24) genes in 20 normal human tissues. Similar to *SLIT2* expression profile, *SLIT3* is expressed strongly in many tissues, while *SLIT1* expression is neuronal specific. We analysed the 5′ CpG island of *SLIT3* and *SLIT1* genes in tumour cell lines and primary tumours for hypermethylation. *SLIT3* was found to be methylated in 12 out of 29 (41%) of breast, one out of 15 (6.7%) lung, two out of six (33%) colorectal and in two out of (29%) glioma tumour cell lines. In tumour cell lines, silenced *SLIT3* associated with hypermethylation and was re-expressed after treatment with 5-aza-2′-deoxycytidine. In primary tumours, *SLIT3* was methylated in 16% of primary breast tumours, 35% of gliomas and 38% of colorectal tumours. Direct sequencing of bisulphite-modified DNA from methylated tumour cell lines and primary tumours demonstrated that majority of the CpG sites analysed were heavily methylated. Thus, both *SLIT2* and *SLIT3* are frequently methylated in gliomas and colorectal cancers, but the frequency of *SLIT3* methylation in lung and breast cancer is significantly less than that for *SLIT2*. We also demonstrated *SLIT1* promoter region hypermethylation in glioma tumour lines (five out of six; 83%), the methylation frequency in glioma tumours was much lower (two out of 20; 10%). Hence, evidence is accumulating for the involvement of members of the guidance cues molecules and their receptors in tumour development.

Epigenetic inactivation of tumour suppressor genes (TSGs) by promoter region CpG island hypermethylation is now well documented and several TSGs have been demonstrated to be inactivated by this mechanism (reviewed in Jones and Baylin, 2002; Herman and Baylin, 2003). In more recent years, a novel class of TSGs has been identified where epigenetic inactivation plays the predominant role, while somatic mutations are rare. This class of genes is exemplified by the 3p21.3 TSG, Ras association domain family 1A gene (*RASSF1A*) (Dammann *et al*, 2000; Lerman and Minna, 2000; Agathanggelou *et al*, 2001; Burbee *et al*, 2001). The CpG island in the promoter region of isoform A is frequently and heavily methylated in many types of cancers, including lung, breast, kidney, NPC, gastric, bladder, neuroblastoma, testicular, etc. (reviewed in Pfeifer *et al*, 2002; Dammann *et al*, 2003), while somatic inactivating mutations are absent or rare.

SLITs, ROBOs and Semaphorins belong to families of proteins that play important roles in axon guidance and cell migration in Drosophila and vertebrates (reviewed in Brose and Tessier-Lavigne, 2000; Wong *et al*, 2002). These proteins are widely expressed in mammalian tissues and the expression is not confined to neurons. Hence, they may have other yet unidentified roles. Slits are secreted proteins that are ligands for the Robo receptors (Brose *et al*, 1999; Kidd *et al*, 1999; Li *et al*, 1999). Recently, Slit was shown to inhibit leucocyte chemotaxis, this inhibition appears to be mediated by Robo (Wu *et al*, 2001). In mammals, four Robo genes have so far been identified, *Robo1*, *Robo2*, Rig-1 (*Robo3*) and magic Robo (*Robo4*) (Kidd *et al*, 1998; Sundaresan *et al*, 1998; Yuan *et al*, 1999; Huminiecki *et al*, 2002). In humans, *ROBO1* is located at 3p12 within a critical region of overlapping homozygous deletions in lung and breast cancers (Sundaresan *et al*, 1998). This region also demonstrated a high frequency of allele loss in lung, kidney and breast cancers. Majority of mice with deletion of exon 2 of *Robo1* die at birth because of delayed lung maturation, the surviving mice develop bronchial hyperplasia (Xian *et al*, 2001). In an earlier study, we demonstrated that there were no inactivating somatic mutations in *ROBO1* in lung and breast cancers, but a CpG island in the 5′ region of *ROBO1* was hypermethylated in breast and kidney tumours (Dallol *et al*, 2002b). We went on to analyse the ligand *SLIT2* located at 4p15.2 for genetic/epigenetic inactivation in tumours. The 4p15.2 region shows frequent allele loss in lung, breast, colorectal and head and neck cancers. *SLIT2* promoter region CpG island was found to be frequently hypermethylated in lung, breast, colorectal and glioma tumours, while somatic mutations were not found (Dallol *et al*, 2002a, 2003a, 2003b). Furthermore, we demonstrated *in vitro* growth suppression when *SLIT2* was expressed in tumour cell lines that had no endogenous *SLIT2* expression due to methylation. A recent paper demonstrated that in mice *slit2* homozygous deficiency was lethal (Plump *et al*, 2002).

In this report, we analysed the methylation status of 5′ CpGs islands for the remaining *SLIT* gene family members (*SLIT3* and *SLIT1*) in human cancers.

## MATERIALS AND METHODS

### Patients and samples

A total of 60 glioma samples, plus seven glioma tumour cell lines (T17, U87-MG, A172, U343, HS683, U373, H4) were analysed for methylation. Among the glioma samples, 40 were classified as gliomblastoma multiforme. The remaining gliomas were collected randomly and consisted of all grades. In addition, 32 colorectal cancer samples and their matching histologically normal mucosa, six colorectal tumour cell lines (SW48, HCT116, LS411, LS174T, DLD1, LoVo), 15 lung tumor cell lines, 32 invasive ductal breast carcinoma plus 29 breast tumour cell lines were also analysed for methylation. These have been described previously (Dallol *et al*, 2002a, 2003a, 2003b)

### Bisulphite modification and methylation analysis

Bisulphite DNA sequencing was performed as described previously (Agathanggelou *et al*, 2001). Briefly, 0.5–1.0 *μ*g of genomic DNA was denatured in 0.3 M NaOH for 15 min at 37°C. Unmethylated cytosine residues were then sulphonated by incubation in 3.12 M sodium bisulphite (pH 5.0) (Sigma, Dorset, UK) and 5 mM hydroquinone (Sigma) in a thermocycler (Hybaid) for 15 s at 99°C and 15 min at 50°C for 20 cycles. Sulphonated DNA was then recovered using the Wizard DNA cleanup system (Promega, Southampton, UK) according to the manufacturers’ instructions. The DNA was desulphonated by addition of 0.3 M NaOH for 10 min at room temperature. The converted DNA was then ethanol precipitated and resuspended in water.

### Combined bisulphite restriction analysis (COBRA) and sequencing

All reactions were performed on a thermocylcer (Hybaid) and with HotStar *Taq* Polymerase (Quiagen, West Sussex, UK). The promoter methylation status of *SLIT3* and *SLIT1* were determined using the COBRA method followed by sequencing to confirm methylation and ascertain the extent of methylation. The cycling conditions were as follows: initial denaturation for 10 min at 95°C, followed by 25–35 cycles of 1 min at 95°C, 1 min at annealing temperature and 2 min at 74°C with a final extension for 5 min at 74°C using the forward and reverse primers. The reaction volume of 20 *μ*l contained 40 ng bisulphate-modified DNA, 1 × PCR Buffer containing 1.5 mM MgCl_2_ (Quiagen), 0.2 M dNTPs, 0.4 *μ*M each primer and 0.5 U HotStar *Taq* (Qiagen). Then, 1 *μ*l of this reaction was used in a seminested PCR reaction (50 *μ*l) using forward and reverse nested primers in the case of *SLIT3* and reverse and forward nested primers for *SLIT1*. The same PCR programme and concentration of reagents were used as before. The annealing temperature, MgCl_2_ concentration and sequences for the gene primers are listed in Table 1

**Table 1:** Expression and methylation primer sequences and PCR conditions

**Primer name**	**Primer sequence (5′ to 3′)**	**Annealing temp. for PCR (°C)**	**MgCl_2_ conc. (mM)**	**Predicted product size (bp)**
*SLIT3* COBRA F	GGT-TAG-TTT-ATT-YGG-TYG-TTT-YGT-G TT-TTA-GT	55	1.5	394
*SLIT3* COBRA R	TAC-CCA-CCC-RAA-AAC-CAT-AAT-ATA-CAA-AA			
*SLIT3* COBRA RN	CCA-CTC-CTA-AAA-AAA-ACT-ACC-TCT-A			
				
*SLIT1* COBRA F	GTT-TAT-TTT-TTT-TTT-YGT-AGT-AGT-TAG-TTG-GGA-GT	57	1.5	370
*SLIT1* COBRA R	TAA-CAA-TCC-ACC-RTA-ATT-CCR-ATA-CAA-ATA-CAA-AAA			
*SLIT1* COBRA FN	GGY-GAA-AYG-GTA-GAG-GAG-TYG-AGT-TTT-T			
				
*GAPDH* expression F	TGA-AGT-TCG-GAG-TCA-ACG-GAT-TTG-GT	60	1.5	982
*GAPDH* expression R	CAT-GTG-GGC-CAT-GAG-GTC-CAC-CAC			
				
*SLST3* expression F	CAA-GTG-TGC-CGA-GGG-CTA-TGG-AG	65	1.5	419
*SLIT3* expression R	ACG-GG C-TTA-GGA-ACA-CGC-GAG-G			
				
*SLIT1* expression F	GTG-ACA-ACT-GCA-GTG-AGA-AC	55	1.5	422
*SLIT 1* expression R	GTA-CAG-CTC-AAC-TGC-AAT-GT			

(Y=C or T and R=A or G). All PCR products were assayed for methylation by incubation with *Bst*UI at 60°C or *Taq*^*α*^I at 65°C for 2 h before visualisation on a 2% agarose gel with added ethidium bromide. The CpG island methylation status for *SLIT3* was determined by cloning PCR products into pGEM T-Easy vector (Promega – according to manufacturers’ instructions). At least five clones from each PCR product were then prepared for sequencing. *SLIT3* Colony PCR products were purified using the QIAquick PCR Purification Columns (Quiagen – according to manufacturers’ instructions) and then reamplified using ABI BigDye Cycle Sequencing Kit (Perkin-Elmer, Warrington, UK) with the reverse nested primer (as shown in Figure 2A). The *SLIT1* COBRA PCR products were purified and then sequenced directly as described above, but with the reverse primer. The reactions were then analysed using an ABI Prism 377 DNA sequencer (Perkin–Elmer).

### Real-time RT–PCR

The theoretical and practical aspects of real-time quantitative RT–PCR using the ABI Prism 7700 Sequence Detection System (Perkin–Elmer) have been described in detail elsewhere (Dallol *et al*, 2002a). Briefly, total RNA was reverse transcribed before real-time PCR amplification. Quantitative values were obtained from the threshold cycle (*C*_t_) number at which the increase in the signal associated with exponential growth of PCR products begins to be detected using the PE Biosystems analysis software, according to the manufacturers’ manuals. The precise amount of total RNA added to each reaction mix and the quality was difficult to assess. We therefore also quantified transcripts of the gene *RPLPO* (also known as 36B4) encoding human acidic ribosomal phosphoprotein PO as the endogenous RNA control, and each sample was normalised on the basis of its *RPLPO* content. Results, expressed as N-fold differences in target gene expression relative to the *RPLPO* gene, termed ‘N*target*’, were determined by the formula: N*target*=2^ΔCt*sample*^, where Δ*C*_t_ value of the sample was determined by subtracting the *C*_t_ value of the target gene from the *C*_t_ value of the *RPLPO* gene. The nucleotide sequences of the primers used for PCR amplification were the following: *SLIT1*-U (5′-CTGGATGGCTTGAGGACCCTAAT-3′) and *SLIT1*-L (5′-GCCCGTGAAGCTGTCGTTGT-3′) with a *SLIT1*-specific product size of 72 bp, *SLIT3*-U (5′-GAATATGTCACCGACCTGCGACT-3′) and *SLIT3*-L (5′-GCAGGTTGGGCAACTTCTTGA-3′) with a *SLIT3*-specific product size of 85 bp, and *RPLPO*-U (5′-GGCGACCTGGAAGTCCAACT-3′) and *RPLPO*-L (5′-CCATCAGCACCACAGCCTTC-3′) with a *RPLPO*-specific product size of 149 bp. PCR was performed using the SYBR® Green PCR Core Reagents kit (Perkin–Elmer). To avoid amplification of contaminated genomic DNA, one of the two primers was placed at the junction between two exons. The thermal cycling conditions comprised of an initial denaturation step at 95°C for 10 min and 50 cycles of 95°C for 15 s and 65°C for 1 min.

### Cell lines and 5-aza-2′ deoxycytidine treatment

Breast, colorectal and glioma tumour cell lines were routinely maintained in RPMI 1640 (Invitrogen, San Diego, CA, USA) supplemented with 10% FCS at 37°C, 5% CO_2_. 5–10 × 10^5^ cells were plated and allowed 24 h growth before addition of 5-aza-2′-deoxycytidine (Sigma). The medium was changed 24 h after treatment and then every 3 days. RNA was prepared at 5 and 7 days after treatment using the Rneasy kit (Qiagen) according to manufacturers’ instructions.

### Expression analysis

Breast, colorectal and glioma cell line were treated with 5 *μ*M demethylating agent 5-aza-2′-deoxycytidine freshly prepared in ddH_2_O and filter-sterilised. Extracted RNA (1 *μ*g) was used as a template for cDNA synthesis using SuperScript™ III RNase H^−^ Reverse Transcriptase (Invitrogen – according to the manufacturers’ instructions). In total, 2 *μ*l (10%) of the first strand reaction was used for PCR (Invitrogen – according to the manufacturers’ instructions). Primers used for *SLIT1*, *SLIT3* and *GAPDH* RT–PCR are described in Table 1. The PCR theromocycle (Hybaid) consisted of an initial denaturation of 10 min at 95°C followed by 35 cycles of 95°C for 30 s, annealing temperature for 30 s, 72°C for 30 s and a final extension of 5 min at 72°C. PCR products were visualised on a 2% agarose gel with added ethidium bromide.

## RESULTS

### *SLIT1* and *SLIT3* expression analysis in normal tissues using quantitative real-time RT–PCR

**Figure 1:**
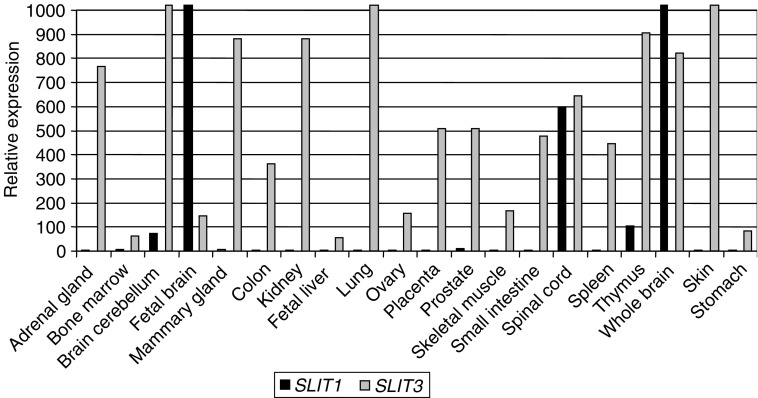
Quantitative real-time RT–PCR was used to measure *SLIT1* and *3* expression levels in 20 normal human tissues. See Materials and Methods for real-time RT–PCR details. *RPLPO* gene transcript was used as the endogenous RNA control and each sample was normalised on the basis of its *RPLPO* content.

We investigated the expression pattern of *SLIT1* and *SLIT3* genes in a wide range of normal human tissues using quantitative real-time RT–PCR (see Materials and Methods). *SLIT3* was expressed in majority of tissues analysed, with the highest expression in skin, brain cerebellum and lung and lowest expression in fetal liver, bone marrow and stomach. While, *SLIT1* expression was much more restricted (brain and nervous system) (Figure 1

).

### Epigenetic inactivation of the *SLIT3* gene in tumour cell lines

**Figure 2:**
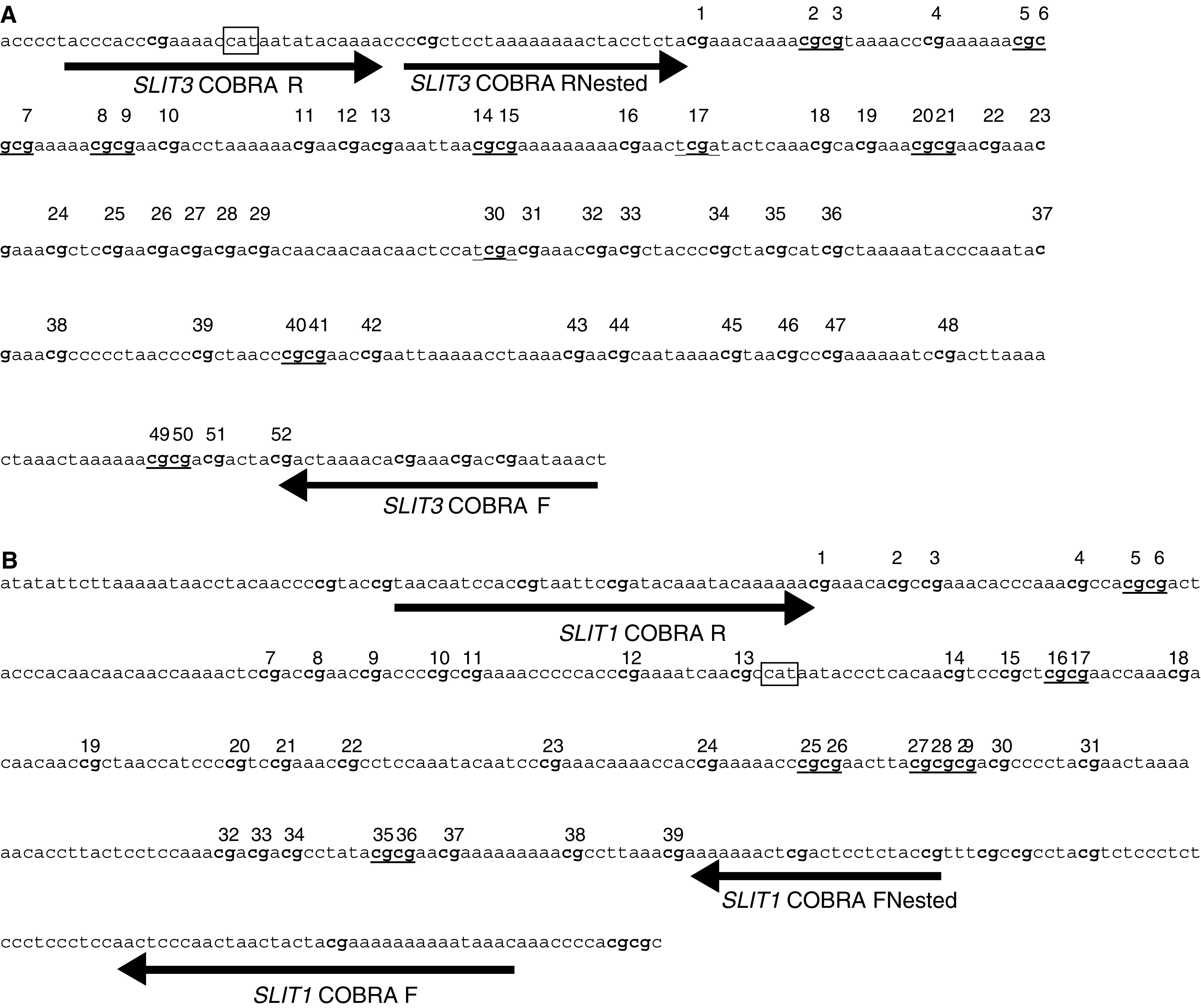
(**A**) Sequence of the reverse bisulphate-modified *SLIT3* promoter region CpG island fragment. Arrows indicate the position of the primers for COBRA. CG dinucleotides within the amplification region are numbered 1–52 and sequences underlined are *Bst*UI and *Taq*I sites used to detect methylation. The expected amplicon size from *SLIT3* COBRA F to *SLIT3* COBRA RNested is 394 bp. The box represents the transcriptional start site of *SLIT3*. (**B**) Sequence of the reverse bisulphate-modified *SLIT1* promoter region CpG island fragment. Arrows indicate the position of the primers for COBRA. CG dinucleotides within the amplification region are numbered 1–39 and sequences underlined are *Bst*UI sites used to detect methylation. The expected amplicon size for *SLIT1* FNested to *SLIT1* R is 370 bp. The box represents the transcriptional start site of *SLIT1*.

**Figure 3:**
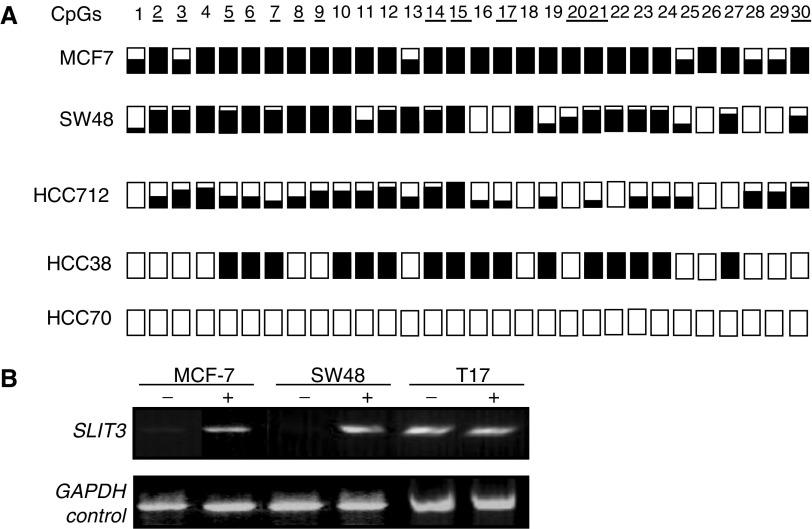
(**A**) *SLIT3* CpG island COBRA PCR products were cloned and sequenced from breast (MCF7, HCC712, HCC38 and HCC70) and colorectal (SW48) tumour cell lines. For each tumour cell line, several clones were sequenced and the methylation status for the first 30 CpGs is shown. White and black squares represent unmethylated and methylated CpGs, respectively. Partially filled squares represent partially methylated CpGs. (**B**) Expression of *SLIT3* in methylated breast (MCF7) and colorectal (SW48) cell lines before and after treatment with the demethylating agent 5-aza-2′-deoxycytidine (5-aza dC). Gene expression was restored by 5-aza-dC (+) in methylated cell lines that lacked *SLIT3* expression. GAPDH and the unmethylated glioma cell line T17 were used as positive controls to ensure RNA integrity and equal loading.

The *SLIT3* putative promoter region was predicted by Promoter Inspector software (http://www.genomatrix.de). This region is from −576 to +9 relative to the translation start site. This region fulfilled the criteria of a CpG island with a GC content of 77% and an observed : expected CpG ratio of 0.86 (CpG plot programme at http://www.ebi.ac.uk/Tools/). We investigated the methylation status of this 5′ CpG island associated with the *SLIT3* gene in various human tumour cell lines. For this analysis, we utilised the COBRA assay on bisulphite-modified DNA. Figure 2A

shows the sequence of the region analysed and the primers used (sequence shown is reverse strand bisulphite modified and all CG methylated), *Taq*I and *Bst*UI sites are underlined. This CpG island was found to be hypermethylated in 12 out of 29 (41%) breast, one out of three (33%) NSCLC, zero out of 12 (0%) SCLC, two out of six (33.3) colorectal and in two out of seven (29%) glioma tumour cell lines. The COBRA PCR products were cloned and several clones of each tumour cell line were sequenced to determine the pattern and extent of methylation. As seen in Figure 3A

, majority of the 30 CG dinucleotides were hypermethylated for MCF-7, HCC712 and HCC38 (breast tumour lines) and SW48 (colorectal tumour line), while breast tumour cell line HCC70 was unmethylated. *SLIT3* expression was restored in tumour lines that were heavily methylated (MCF-7 and SW48) by treating the cell lines with 5aza-2-deocycytidine (Figure 3B), while the unmethylated glioma tumour cell line T17 did not show any change in expression.

### Epigenetic inactivation of the *SLIT3* gene in primary tumours

**Figure 4:**
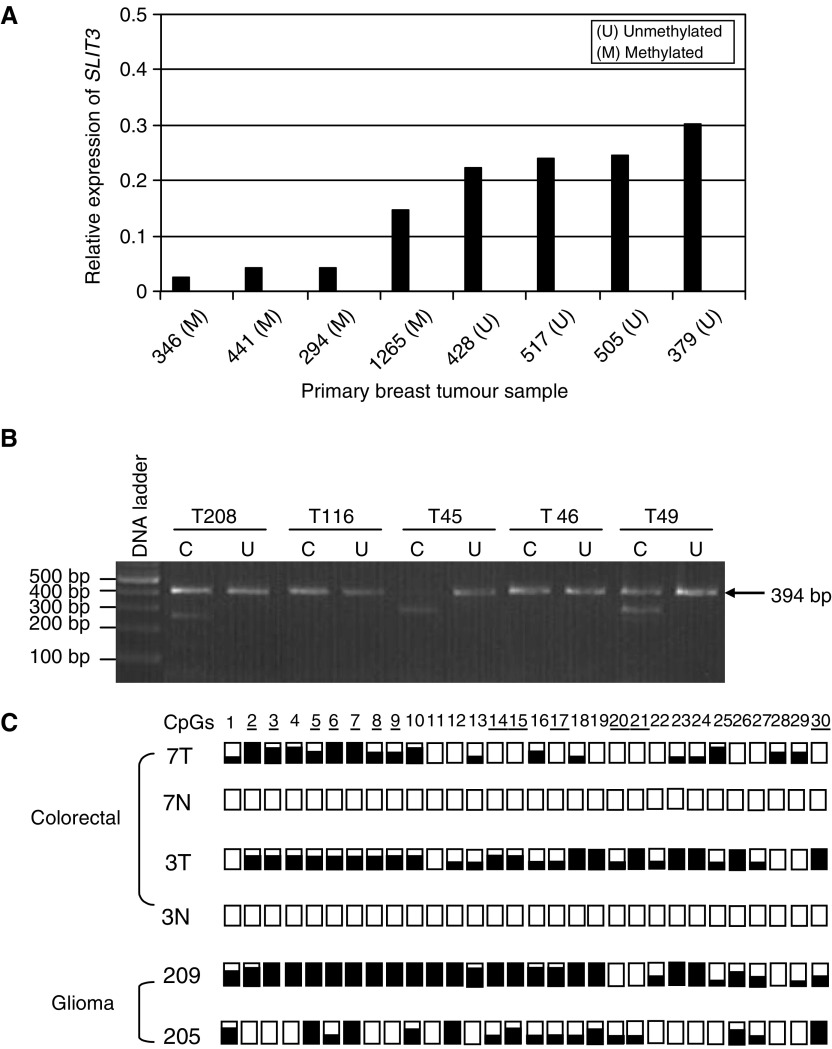
(**A**) Relative expression of *SLIT3* gene in methylated (M) and unmethylated (U) breast tumours using real-time RT–PCR as described in Materials and Methods (**B**) *Bst*UI digest of *SLIT3* CpG island COBRA PCR products from glioma (T208, T116, T45) and colorectal (T46, T49) primary tumours. (**C**) COBRA PCR products from glioma and colorectal primary tumours were cloned and sequenced. For each tumour, several clones were sequenced and the methylation status for the first 30 CpGs is shown. White and black squares represent unmethylated and methylated CpGs, resepectively. Partially filled squares represent partially methylated CpGs. 7T and 3T represent colorectal tumours, 7N and 3N represent corresponding normal colon samples. 209T and 205T represent glioma tumours.

We then analysed the methylation status of the above CpG island in primary tumours using COBRA analysis followed by sequencing of the cloned PCR products. Five out of 32 (16%) breast tumours were found to be methylated for *SLIT3*. Using real-time RT–PCR, we demonstrated that the *SLIT3* methylation in breast tumours correlated with reduced *SLIT3* expression as compared to unmethylated breast tumours (Figure 4A

). *SLIT3* 5′ CpG island was also hypermethylated in 12 out of 32 (37.5%) colorectal tumours and in 21 out of 60 (35%) glioma primary tumours. Since the tumour samples used in this study were not microdissected, in majority of the primary tumors unmethylated bands were also detected. No methylation was found in corresponding normal tissues from the colorectal or glioma patients or in the DNA isolated from normal brains. Similar to the tumour cell line data, sequencing of cloned PCR products confirmed that majority of the 30 CG dinculeotides analysed were methylated in primary tumours (Figure 4B, C).

### Epigenetic inactivation of *SLIT1* gene in gliomas

**Figure 5:**
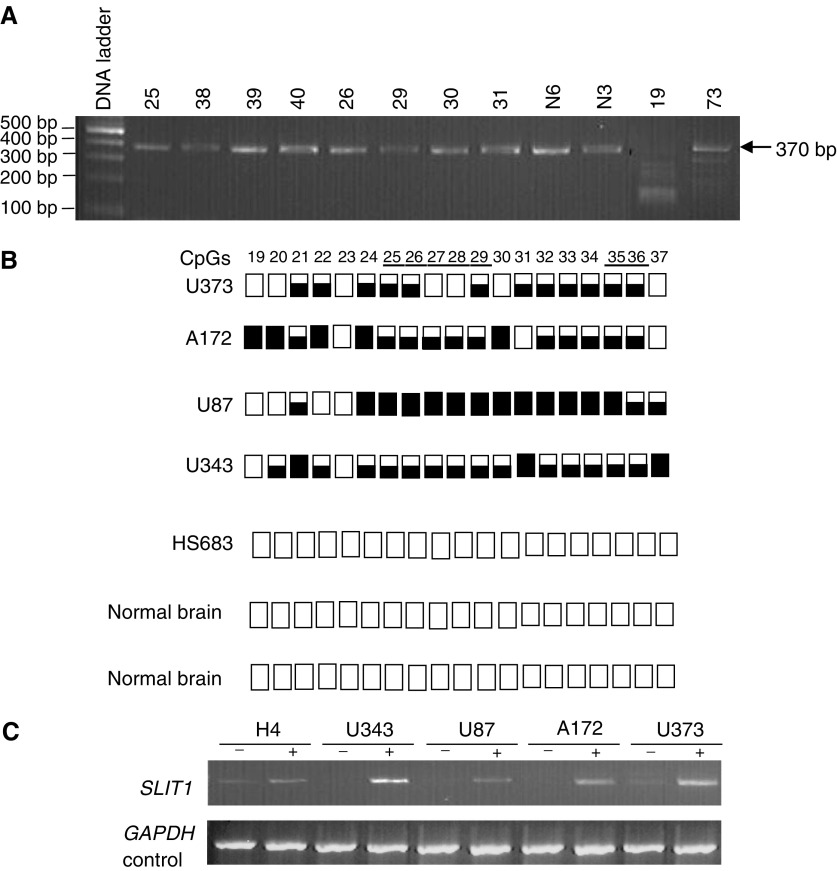
(**A**) *Bst*UI digest of *SLIT1* COBRA PCR products from glioma grade IV tumours and normal brain (N6 and N3) which were included as negative controls for methylation. Tumours 19 and 73 show digestion with B*st*UI. (**B**) *SLIT1* COBRA PCR products were directly sequenced from glioma tumour cell lines and normal brain. The methylation status from CpG 19–37 is shown. White and black squares represent unmethylated and methylated CpGs, respectively. Partially filled squares represent partially methylated CpGs. Glioma tumour cell line Hs683 is unmethylated for *SLIT1* (**C**) Expression of *SLIT1* in glioma tumour cell lines before and after treatment with 5-aza-2′-deoxycytidine. GAPDH was used as positive control to ensure RNA integrity and equal loading.

The *SLIT1* CpG island was predicted to be from −574 to +192 and had a GC content of 71% and had an observed : expected CpG ratio of 0.81 (CpG plot programme at http://www.ebi.ac.uk/Tools/). This region overlapped with the *SLIT1* putative promoter region predicted by Promoter Inspector software (http://www.genomatrix.de). Since *SLIT1* expression is neuronal specific, we analysed the methylation status of the 5′ CpG island of the *SLIT1* gene in glioma tumour cell lines and primary tumours by COBRA and direct sequencing of bisulphite-modified DNA (Figure 2B). Five out of six (83%) glioma tumour lines were methylated, while only two of 20 (10%) glioma tumours demonstrated *SLIT1* methylation (Figure 5A, B

). No methylation was found in DNA isolated from normal brains. *SLIT1* expression was restored/upregulated in five glioma tumour lines (methylated for *SLIT1)* by treatment with 5-aza-2′-deoxycytidine (Figure 5C).

## DISCUSSION

The Slit genes encode ligands for the roundabout (robo) receptors. The Slit–Robo interactions mediate the repulsive cues on axons and growth cones during neural development. The Slit family comprises of large extracellular matrix-secreted and membrane-associated glycoproteins with multiple functional domains (reviewed in Brose and Tessier-Lavigne, 2000). Slit genes have been identified in Drosophila, *Caenorhabditis elegans*, Xenopus, chickens, mice, rats and humans. There are three known mammalian *SLIT* genes (*SLIT1*, *SLIT2*, *SLIT3*) located on chromosome 10q23.3–q24, 4p15.2 and 5q35–q34, respectively. *slit2* and *slit3* genes are expressed in neuronal as well as nonneuronal tissues, while *slit1* expression is specific to the brain (Wu *et al*, 2001 and this report). *Slit2* homozygous deficiency in mice is lethal, while *Slit1* and *Slit3* homozygous mice are viable (Plump *et al*, 2002; Yuan *et al*., 2003).

In our earlier studies, we demonstrated that the ligand (*SLIT2*) for *robo1* receptor was frequently methylated in lung, breast, colorectal and glioma tumours and that the methylation correlated with loss of *SLIT2* expression. More recently, we demonstrated *SLIT2* methylation in neuroblastoma, Wilms’ tumour and renal cell carcinoma (Astuti *et al*, 2004). Furthermore, in *in vitro* assays, *SLIT2* suppressed tumour growth (Dallol *et al*, 2002a, 2003a, 2003b).

*SLIT3* is located at 5q35-q34, which is a frequent region of allelic loss in colorectal and lung cancers (Girard *et al*, 2000; Goel *et al*, 2003). We have now demonstrated that *SLIT3* 5′ CpG island similar to *SLIT2* is frequently hypermethylated in colorectal and glioma tumours and less so in breast tumours. And loss of *SLIT3* expression can be reversed by treatment with a demethylating agent. While *SLIT1* gene is frequently methylated in glioma tumour lines but at low frequencies in glioma tumours, hence *SLIT1* may play a role in late gliomagenesis.

Slits, netrins, semaphorins and the ephrins constitute conserved families of axonal guidance cues that have prominent developmental effects. Recently, *SEMA3B* was also demonstrated to be inactivated in lung cancer by promoter region hypermethylation (Tomizawa *et al*, 2001; Kuroki *et al*, 2003). Re-expression of SEMA3B inhibited lung cancer cell growth and induced apoptosis.

The finding of epigenetic inactivation in various human cancers of *ROBO1*, *SEMA3B*, *SLIT2* and now *SLIT3* and to a lesser extent *SLIT1*, all of which are involved in axon and cell migration in Drosophila and vertebrates, suggests a novel, and common underlying theme for these molecules in tumour suppression.
